# Protective roles of intra-arterial mild hypothermia and arterial thrombolysis in acute cerebral infarction

**DOI:** 10.1186/s40064-016-3654-7

**Published:** 2016-11-17

**Authors:** Xiaoxiang Peng, Yue Wan, Wenjun Liu, Bitang Dan, Li Lin, Zhouping Tang

**Affiliations:** 1 Department of Neurology, Tongji Medical College, Huazhong University of Science and Technology, Wuhan, 430074 China; 2 Department of Neurology, Wuhan Third Hospital, Wuhan, 430074 China

**Keywords:** Mild hypothermia, Acute cerebral infarction, Arterial thrombolysis, Cerebral protection

## Abstract

**Objective:**

Herein, we evaluated the efficacy and safety of intra-arterial mild hypothermia in combination with arterial thrombolysis to treat acute cerebral infarction due to middle cerebral artery occlusion.

**Methods:**

A total of 26 patients with acute middle cerebral artery occlusion were divided into a normothermia group (n = 15) and a mild hypothermia group (n = 11). The infarct volumes at 24 h and 7 days after the operation were compared between the normothermia group and the mild hypothermia group. Additionally, we compared neurological deficit scores between the two groups at 24 h, 7 days, and 1 mo after the operation.

**Results:**

The infarct volumes and neurological deficit scores of the mild hypothermia group were significantly reduced compared to those in the normothermia group (*p* < 0.05). Furthermore, no adverse reactions or complications occurred in the mild hypothermia group.

**Conclusion:**

Intra-arterial mild hypothermia reduced infarct volume after ischemia–reperfusion injury in the arterial thrombolysis of an acute cerebral infarction. Additionally, it improved the prognosis of patients with an acute middle cerebral artery occlusion, suggesting that this procedure is safe and effective for treating acute cerebral infarction.

## Background

Ischemic cerebrovascular disease, which is otherwise known as a stroke, is the second leading global cause of mortality after coronary heart disease and a major cause of neurological disability (Lopez et al. 2006). Approximately 17 million strokes occur worldwide each year (Feigin et al. 2014), and stroke is not only a life-threatening disease, but also a recurrent disease (Birns and Bhalla 2015). Following the acute phase, stroke patients often require long-term rehabilitation and nursing home care with ongoing support from their community (Mikulik and Wahlgren 2015). Thus, stroke is a devastating disease and a major economic burden on society (Mounica 2015). Moreover, the quality of life of stroke patients generally remains poor (van Eeden et al. 2015).

Ischemic cerebrovascular disease that involves an acute middle cerebral artery occlusion is associated with higher rates of disability and mortality (Gawlitza et al. 2016). Typically, for such a condition, early stage intravenous thrombolysis is performed (The National Institute of Neurological Disorders and Stroke rt-PA Stroke Study Group 1995) and the drug alteplase, a tissue plasminogen activator, is administered (Campbell et al. 2015a). This is the only therapy with a proven efficacy for acute ischemic stroke when used within 3–5 h after stroke onset (Fisher and Saver 2015). However, because of the complexity of intravenous thrombolysis (Shao et al. 2015), little improvement has been made in the use of thrombolysis procedures for acute stroke (Marshall 2015).

The multicenter randomized clinical trial of endovascular treatment for acute ischemic stroke in the Netherlands (MR CLEAN) reported on the efficacy of intra-arterial (IA) treatment for acute ischemic stroke (AIS) (Rozeman et al. 2016). Their results indicated that IA therapy is beneficial for patients with an acute middle cerebral artery occlusion. Arterial thrombolysis can promptly restore blood supply to ischemic brain tissue and protect the brain tissue within the penumbra. However, arterial thrombolysis can also trigger ischemic-reperfusion injury; therefore, intra-arterial thrombolysis must be carried out within a strict time frame. Ischemia–reperfusion injury is a major cause of post-stroke impairment, which seriously affects the quality of life and the functional rehabilitation of stroke patients (Liu et al. 2015).

Induced hypothermia has been used to prevent or reduce ischemia–reperfusion injury. For example, it has been shown that a combination of mild hypothermia and sodium hydrosulfide treatment is beneficial for reducing hippocampal apoptosis and pathology (Dai et al. 2016). In another study, deep hypothermia and diazoxide, a potassium channel activator, were correlated with a reduction in DNA fragmentation and the inhibition of mitochondrial cytochrome c release and caspase-3 activation (He et al. 2008). Further, there have been 5 randomized controlled trials (RCTs) showing the benefit of intra-arterial endovascular treatment for acute ischemic stroke (MR CLEAN, REVASCAT, ESCAPE, SWIFT PRIME, EXTEND-IA) (Berkhemer et al. 2015; Campbell et al. 2015b; Goyal et al. 2015; Jovin et al. 2015; Saver et al. 2015).

Most stroke patients are referred to specialists too late for optimal arterial thrombolysis. Thus, we here evaluated the utility of intra-arterial mild hypothermia combined with arterial thrombolysis for treating acute middle cerebral artery occlusion.

## Methods

### Subjects

We selected patients based on five criteria. First, patients clinically diagnosed with an acute middle cerebral artery occlusion and disease onset of less than 6 h were included. Second, patients with early signs of cerebral hemorrhage and cerebral infarction shown on a cranial computed tomography (CT) scan were excluded. Third, we restricted the age of the patients to between 18 and 75 years, inclusive. Fourth, only patients without severe organ dysfunction or a bleeding tendency were included. Finally, we required that the etiology of the cerebral infarct of the patients was atherosclerosis. Patients with a disease onset duration of more than 6 h were excluded from surgery of the vascular occlusion. This study was conducted in accordance with the declaration of Helsinki. This study was conducted with approval from the Ethics Committee of Huazhong University of Science and Technology. Written informed consent was obtained from all participants. The protocol number of the study was QJX2010-43.

### Grouping

A total of 26 patients with an acute middle cerebral artery occlusion were randomly divided into a normothermia group (n = 15) and a mild hypothermia group (n = 11). All 26 patients with acute MCA (16 males, 10 females). Patients in the mild hypothermia group received arterial thrombolysis combined with intra-arterial mild hypothermia. To induce neuroprotection, we used a partial mild hypothermia temperature range of 28–33 °C. The patients in the conventional treatment group had met the inclusion criteria but were either unsuitable for interventional therapy, or their legal guardian rejected the therapy.

### Operation

The vascular occlusion site in each patient was confirmed using conventional cerebral angiography under anesthesia. Using a digital subtraction angiography system, we inserted the microguidewire (PT Graphix™, Boston Scientific, Marlborough, MA, USA) with a microcatheter (Rebar™-18, ev3, Plymouth, MN, USA) through the thrombus. Then, the microguidewire was pulled out and the angiography was performed through the inserted microcatheter. After confirmation that the distal vascular was removed, Ringer’s solution (50 mL/min, total 500 mL) at 4 °C was pumped into the brain tissue through the microcatheter over 10 min so that the temperature of the local brain tissue was quickly reduced to induce mild hypothermia. The Ringer’s solution consisted of 120 mM NaCl, 25 mM NaHCO_3_, 3.3 mM KCl, 1.2 mM NaH_2_PO_4_, 1.8 mM CaCl_2_, 2.4 mM MgSO_4_, and 10 mM dextrose, and was percolated with 95% O_2_ and 5% CO_2_ before use. Subsequently, super-selective thrombolysis with urokinase was carried out through the inserted microcatheter in combination with balloon dilatation (Maverike2™, Boston Scientific), stent embolectomy (Solitaire™ FR, ev3), or implantation of the stent into a blood vessel to restore blood flow.

### Neurological deficit scoring

Neurological deficit scoring according to the National Institutes of Health Stroke Scale scoring method was performed at 24 h, 7 days, and 1 mo after the operation.

### Determination of infarct volume

The infarct sizes of each patient were determined at 24 h and 7 days after the operation using cranial magnetic resonance imaging (MRI) (Philips, Eindhoven, Netherlands) and diffusion weighted imaging. A 3.0 Tesla MRI scan was performed using a susceptibility weighted protocol that was optimized for brain imaging. The infarct volumes were calculated using the Streiner formula.

### Determination of clinical indexes

The blood pressure, heart rate, blood glucose, blood gas, and coagulation condition of each patient were recorded 24 h after the operation.

### Statistical analysis

SPSS statistical software (version 11.5; IBM Corporation, Armonk, NY, USA) was used to analyze the data. Measurement data are reported here as the mean ± standard deviation (SD) and groups were compared using a *t* test. Numeration data were compared using a Chi squared test. For all of the results, p values less than 0.05 were considered statistically significant.

## Results

### Infarct volumes after induced intra-arterial mild hypothermia

**Fig. 1:**
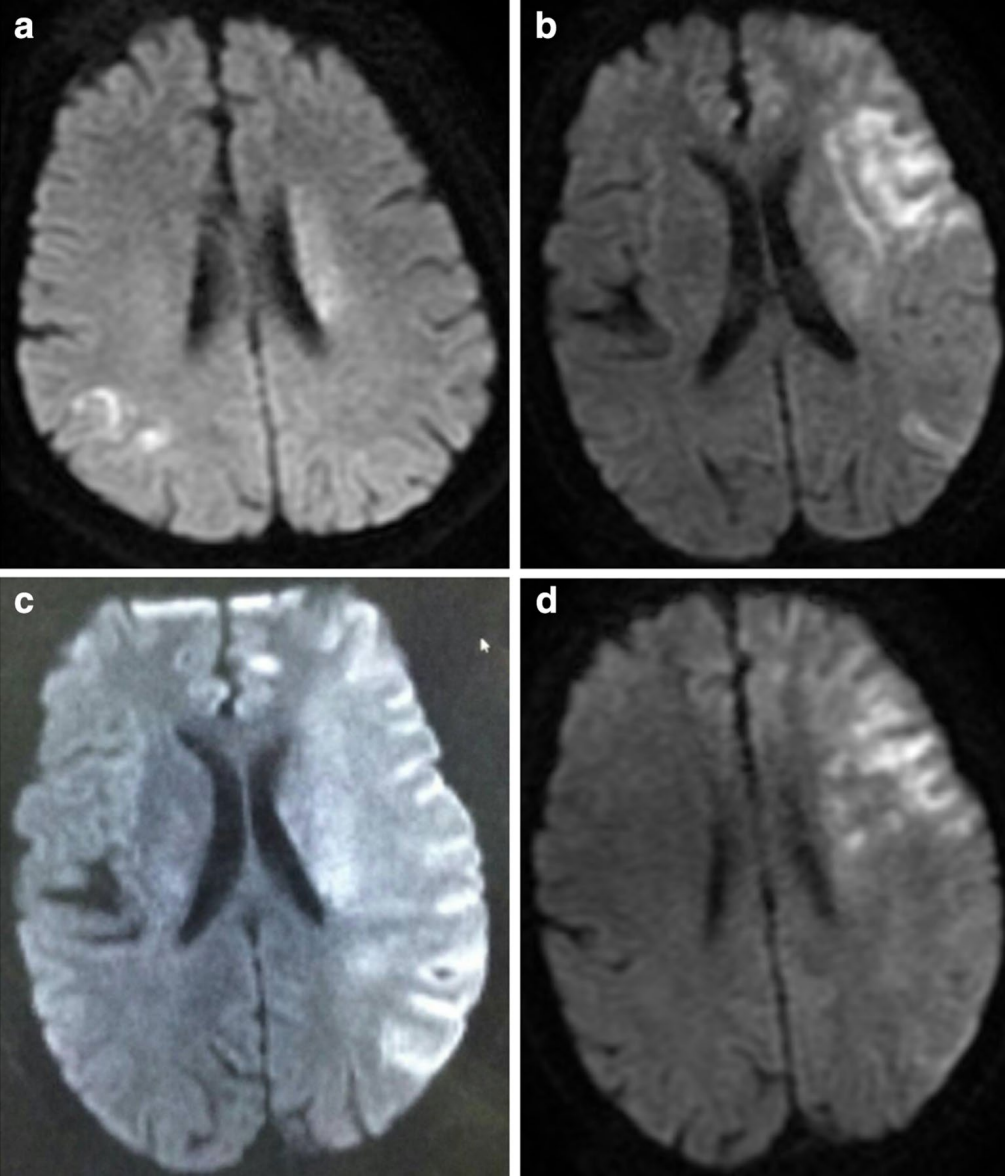
The representative MRIs were presented in the normothermia group and mild hypothermia group. **a** The representative MRI of preoperative in the normothermia group. **b** The representative MRI of postoperation 1 week in the normothermia group. **c** The representative MRI of preoperative in the mild hypothermia group. **d** The representative MRI of postoperation 1 week in the mild hypothermia group

The representative MRIs were presented in the normothermia group and mild hypothermia group (Fig. 1). The average pre-treatment infarct volumes in the normothermia and mild hypothermia groups were 25.03 ± 10.12 and 24.91 ± 9.35 mm^3^, respectively. The average infarct volume in the mild hypothermia group was 13.45 ± 6.01 mm^3^ 24 h after the operation and 12.25 ± 7.42 mm^3^ 7 days after the operation. The average infarct volume in the mild hypothermia group was significantly smaller than that in the normothermia group at both 24 h and 7 days after the operation (*p* < 0.05; Table 1).

**Table 1:** The infarct volumes (mm^3^) in the two groups at different time points (mean ± SD)

Groups	The infarct volumes in pre-treatment	The infarct volumes after the operation time	
	0 h	24 h	7 days
Normothermia	25.03 ± 10.12	25.56 ± 10.22	26.26 ± 10.86
Mild hypothermia	24.91 ± 9.35	13.45 ± 6.01*	12.25 ± 7.42*

* *P* < 0.05 versus the normothermia group

### Neurological scores after intra-arterial mild hypothermia

The neurological deficit scores were 16.75 ± 8.24 and 16.25 ± 8.51 in the normothermia group and mild hypothermia group, respectively. The mean neurological deficit scores for the mild hypothermia group were 10.04 ± 6.85 at 24 h, 9.06 ± 6.20 at 7 days, and 7.06 ± 5.13 at 1 mo. The neurological scores in the mild hypothermia group prominently improved at 24 h, 7 days, and 1 mo after the operation compared with those scores in the normothermia group (*p* < 0.05; Table 2).

**Table 2:** The neurological scores in the two groups at different time points (NIHSS)

Groups	The neurological scores after the operation time		
	24 h	7 days	1 m
Normothermia	12.26 ± 5.53	11.65 ± 5.87	9.21 ± 3.43
Mild hypothermia	10.04 ± 6.85*	9.06 ± 6.20*	7.06 ± 5.13*

* *P* < 0.05 versus the normothermia group

### Clinical characteristics of the patients after both treatments

The blood pressure, heart rate, blood glucose, blood gas, and coagulation conditions of the patients in the two groups were recorded at 24 h after the operation and compared. No significant changes in these clinical characteristics were observed between the two groups (*p* > 0.05; Table 3). There was no significant difference regarding complications between the two groups. Included in this analysis were embolization into new territories outside the target downstream territory of the occluded vessel in 2 of the 26 patients (7.7%) and procedure-related vessel dissections in 1 patient (3.8%).

**Table 3:** The clinical characteristics of the patients in the two groups at 24 h after the operation

Groups	Blood pressure (mmHg)	Heart rate (times/min)	Blood glucose (concentration, mmol/l)	Blood gas (mmHg)
Normothermia	132 ± 6.27/72 ± 5.15	75 ± 5.06	6.17 ± 2.48	92.4 ± 5.12
Mild hypothermia	138 ± 9.27/80 ± 8.35	82 ± 7.80	7.82 ± 2.73	93.1 ± 5.89

*P* > 0.05 versus the normothermia group

## Discussion

Middle cerebral artery occlusion generally leads to a massive cerebral infarction, which has a poor prognosis that includes severe disability and death. Thus, timely recovery of blood flow into the infarct area is critical for these patients. The recanalization rate for intravenous thrombolysis of a middle cerebral artery occlusion is low; thus, intra-arterial thrombolysis has been increasingly applied in clinical practice to greatly improve the recanalization rate. A retrospective study of 350 patients with acute stroke undergoing a variety of endovascular treatment techniques found that angioplasty combined with intra-arterial thrombolysis can greatly improve the recanalization rate (Brekenfeld et al. 2005). Among our 22 patients undergoing thrombolysis and angioplasty, 19 patients were completely recanalized and only 3 patients were partially recanalized. The high recanalization rate in our study is likely due to our intra-vascular technique and strict patient inclusion criteria.

Since the first use of mild hypothermia induction for clinical treatment by the American neurosurgeon Fay in 1938, clinical studies of mild hypothermia have been successively reported. Since the 1980s, several studies on the application of mild hypothermia for cerebral ischemia have been carried out (Baker et al. 1991; Ridenour et al. 1992; Zausinger et al. 2000). These studies have shown that the induction of mild hypothermia between 28 and 33 °C has neuroprotective effects. In the clinic, ice blankets, ice caps, and alcohol sponge baths are often used to reduce body temperature. At the International Stroke Conference in 2010, a preliminary report of a clinical trial using endovascular hypothermia combined with intravenous thrombolysis for the treatment of acute ischemic stroke showed that mild hypothermia treatment is safe and feasible (Hemmen et al. 2010). Another study used mild hypothermia combined with interventional arterial thrombolysis for the treatment of acute ischemic stroke and achieved the desired cerebral protective effects (Ding et al. 2002). Subsequently, numerous studies have confirmed that mild hypothermia after stroke can protect brain structure and function at the cellular level (De Georgia et al. 2004; Hemmen and Lyden 2007; Krieger et al. 2001; Zausinger et al. 2003; Zhang et al. 2009). One meta-analysis of 101 animal experiments showed that mild hypothermia can simultaneously reduce infarct volume by 44% on average and increase neurobehavioral scores by 46% (van der Worp et al. 2007). Subsequent experiments demonstrated that localized mild hypothermia combined with intra-arteriovenous thrombolysis is highly effective (Chen et al. 2013; Choi et al. 2010; Guluma et al. 2006; Hong et al. 2014; Konstas et al. 2007; Piironen et al. 2014; Zhao et al. 2009). Here, we found that the average infarct volume decreased from 25 to 13 cm^3^ in the first 24 h for the hypothermia group, which was nearly a 50% volume reduction. Several experimental studies have confirmed that the therapeutic effects of mild hypothermia occur through blocking of reperfusion injury. In the early stage of a cerebral infarction, mild hypothermia therapy can reduce brain tissue injury in the ischemic penumbra and improve cerebrovascular autoregulation, thereby reducing neuronal cell death. In addition, mild hypothermia can protect the blood–brain barrier, reduce cerebral edema, and decrease the influx of calcium to block its neurotoxic effects (Krieger and Yenari 2004; Rogalewski et al. 2006). In addition to the significant infarct volume reduction, average NIHSS scores at various time points were roughly 2 points lower in the hypothermia group. There was a statistically significant difference between the 2 groups. The clinical symptoms improved have three time points, and infarct volume has three time points. The average NIHSS scores for the hypothermia group at 24 h and 7 days after surgery were lower than the average pre-operation score. Although the NIHSS scores were very close at 7 days post-operation, we confirmed that the scores were true and accurately measured. The present study used a small sample size; therefore, the results could not be further analyzed by stratification of the ischemia and reperfusion duration after mild hypothermia and thrombolysis treatment. Thus, future studies should continue to investigate the therapeutic efficacy of mild hypothermia combined with intra-arteriovenous thrombolysis after cerebral infarction.
